# Specific body mass index trajectories were related to musculoskeletal pain and mortality: 19‐year follow‐up cohort

**DOI:** 10.1016/j.jclinepi.2021.09.020

**Published:** 2022-01

**Authors:** Maja R. Radojčić, Romain S. Perera, Lingxiao Chen, Tim D. Spector, Deborah J. Hart, Manuela L. Ferreira, Nigel K. Arden

**Affiliations:** a Nuffield Department of Orthopedics, Rheumatology and Musculoskeletal Sciences, University of Oxford, Oxford, United Kingdom; b Centre for Sport, Exercise and Osteoarthritis Research Versus Arthritis, University of Oxford, Oxford, United Kingdom; c Department of Allied Health Sciences, Faculty of Medicine, University of Colombo, Colombo, Sri Lanka; d Faculty of Medicine and Health, Institute of Bone and Joint Research, The Kolling Institute, University of Sydney, Sydney, Australia; e Department of Twin Research and Genetic Epidemiology, King's College London, London, United Kingdom

**Keywords:** Body mass index, Group-based trajectory modelling, Musculoskeletal pain, Knee pain, Back pain, Mortality

## Abstract

**Objective:**

We aimed to study 19-year body mass index (BMI) patterns and their (1) bidirectional relationship with musculoskeletal pain and (2) mortality risk.

**Study design and Setting:**

We used data from the Chingford study and group-based trajectory modelling to define 19-year BMI patterns. We investigated whether baseline back, hand, hip, and knee pain (as single- and multi-site) predicted 19-year BMI trajectory, and whether 19-year BMI patterns predicted pain in year 20. We explored BMI trajectories and mortality risk over 25 years (life expectancy).

**Results:**

We included 938 women (mean age: year-1=54, year-20=72) and found seven distinct 19-year BMI trajectories: two normal-weighted (reference), slightly overweight, lower and upper overweight-to-obese, lower and upper obese. BMI patterns capturing the increase overweight-to-obese (BMI 27-34 overtime) were bidirectionally related to knee and multi-site pain. The lower obese pattern (BMI 33-38) was unidirectionally associated with lower limb pain. Women with BMI above 40 had an increased all-cause and cardiovascular mortality risk.

**Conclusion:**

For most postmenopausal women, the overweight WHO category was a transition. Two patterns capturing increase overweight-to-obese were mutually related to musculoskeletal pain, i.e., knee and multi-site pain contributed to becoming obese, and trajectories of becoming obese increased the odds of experiencing pain later.

## Highlights

• Different latent subgroups of 19-year body mass index (BMI) patterns exist in UK postmenopausal women.
• BMI of 27-34 over 19-years was bidirectionally related to knee and multi-site pain.
• BMI of 33-38 over time was unidirectionally associated with hip and knee pain.
• Women with BMI>40 had increased all-cause and cardiovascular mortality risk.
• Women with BMI 25-27 had no different pain or mortality risk than women with BMI<25.

## Graphical_abstract

**Figure:**
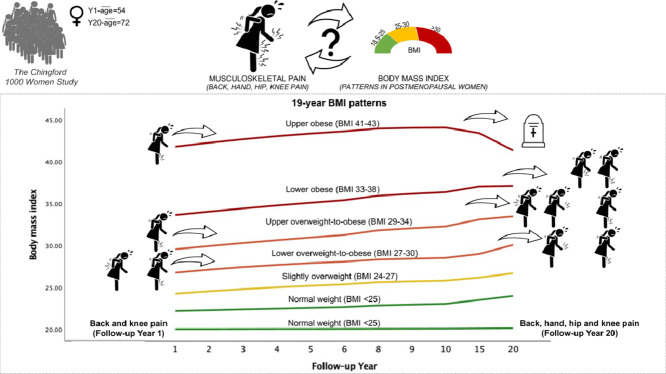

**What is new?**

• Different latent subgroups of 19-year BMI patterns were observed in UK postmenopausal women.
• BMI patterns capturing transition overweight-to-obese and pain in the knee and multi-site mutually influenced each other over time.
• 19-year lower obese trajectory (BMI 33-38) was later related to lower limb (knee and hip) pain.
• Women with upper obese trajectory (BMI above 40) were at an increased risk of all-cause and cardiovascular mortality.
• Slightly overweight women (BMI 25-27) had no different risk of pain or mortality than normal-weighted women; thus, prevention, and non-pharmacological management of musculoskeletal pain may want to target BMI below 27.

## 1 Introduction

Obesity is a global problem related to numerous non-communicable diseases and mortality. According to the World Health Organization (WHO), in 2016, there were 1.9 billion (39%) overweight, and over 650 million (13%) obese adults in the world [1]. The global, European, and United Kingdom (UK) estimates showed that obesity was more prevalent in women [2]. Body mass index (BMI), used for defining overweight and obesity, has been associated with musculoskeletal disorders [1], which account for significant disability-adjusted life-years (DALYs) in non-communicable diseases [3], [4], [5]. Among musculoskeletal disorders, between 1990-2010, back pain accounted for the most DALYs, although osteoarthritis (OA) had the most rapid increase in disease burden [3]. OA often affects knees, hips and hands, and it is the most common cause of pain in the joints [6]. However, musculoskeletal pain is the primary determinant of patients’ impaired quality of life, and delivery of pharmacological or surgical treatments [7].

The association between BMI and musculoskeletal pain has been traditionally researched using one-time BMI measure to cross-sectionally and longitudinally relate it to pain outcomes [8], [9], [10]. Its significance has been recognizing the association and advising overweight/obese patients on weight reduction. However, BMI can change over time, have a cumulative effect, and the relationship between BMI and pain may be bidirectional. Namely, as BMI over time can be related to musculoskeletal pain, likewise, the experience of pain can be associated with a specific BMI pattern, possibly reflecting the change due to advised weight reduction or pain-related decreased quality of life. There has been only one report that used longitudinal, 10-year trajectories of BMI change concerning knee pain, in Japanese women [11]. Yet, there is no evidence whether the experience of musculoskeletal pain is related to any BMI pattern in future.

Thus, given the epidemiological parameters, the global burden and paucity of research, we aimed to investigate BMI trajectories over 19 years in a sample of middle-aged women representative of the UK population and to assess their bidirectional relationship with single- and multi-site musculoskeletal pain and the risk of mortality.

## 2 Methods

### 2.1 Study sample

The Chingford 1,000 women study is a prospective population-based cohort of women 45-64 years old at the study set up (1989), registered in the large general practice in Chingford (North East London, United Kingdom) [12]. All registered women were contacted, and 1,003 (78% response rate) participated at the baseline visit [13]. Over time, women have taken part in different examinations aiming to understand common medical conditions. For this study, we used repeated BMI measures, musculoskeletal pain, and mortality data. The Chingford study has been recognized as a representative sample of women in the United Kingdom [14].

The Waltham Forest and Redbridge local research ethics committee approved the study, and all participants have provided their written informed consents for participation.

### 2.2 Body mass index

We calculated BMI in kg/m^2^ using weight and height measures. The measures were taken barefoot with indoor clothes using an electronic scale for bodyweight and a wall-mount stadiometer (Seca Leicester Height Measure, Birmingham, United Kingdom) for height to the nearest 0.1 kg and 0.1 cm [8,12]. These were available at years 1, 2, 3, 4, 5, 6, 8, 9, 10, 15 and 20, in total, 11 repeated measures of continuous BMI variables.

### 2.3 Musculoskeletal pain

We used musculoskeletal pain assessed at years 1 and 20. Year 1 variables were used as predictors and year 20 variables as distal outcomes. The baseline assessment included back pain (ever experienced an episode lasting more than a week), current hand (the presence of pain in any interphalangeal joint, left or right) and current (left or right) knee pain. In year 20, musculoskeletal pain was available for four sites: back, hand, hip, and knee. The back pain question assessed pain lasting most days per month in the previous three months. Hand, hip, and knee pain variables defined the last month presence of pain in either left or right joints. All pain variables were binary, self-reported in the questionnaire administered by a nurse.

Using the single-site pain reports, we defined binary variables of multi-site musculoskeletal pain. It aimed to address the severity of spread at each follow-up. Multi-site pain variables defined the specified number of affected sites referenced to less affected sites and no pain. For descriptive purposes, we showed each combination of two or three affected sites, however, for the analyses, we used variables corresponding to any two, any/all three, and all four affected sites.

### 2.4 Mortality

We received information on all-cause and cause-specific – cardiovascular and cancer – mortality from the Health and Social Care Information Centre in August 2014 [15]. The Chingford study women were linked to the Office for National Statistics that provided the cause of death information from the death certificates. These data were available over 25 years of the follow-up and used for time-to-event analysis.

### 2.5 Covariates

We used self-reported year 1 and 20 variables biologically related to BMI, musculoskeletal pain, and mortality in women as sample descriptors and confounding variables. Age was a continuous measure. Binary variables were: current menopause status, physical activity, ever used oral contraceptive pill or hormone replacement therapy, current analgesic medication use, ever had hysterectomy, cancer, orthopedic operations, other major illness (including diseases of the cardiovascular, nervous, respiratory, alimentary, endocrine, and urogenital system) and any fractures in last ten years. Physical activity summarized walking, sport, and job-related activity. In year 1, walking >5 miles, sport >2 hours per week and jobs that assumed activity at least half of the working time indicated activity. At year 20, women were active if reporting at least three days per week walking, light, moderate, strenuous sport or muscle strengthening and job involving standing/walking, heavy housework and gardening. The current analgesics use included medications directly or indirectly affecting pain, i.e., Anatomical Therapeutical Chemical classes H02-systemic corticosteroids, M01-anti-inflammatory and antirheumatic products, M02-topical products for joint and muscular pain, M03-muscle relaxants, M04-antigout preparations and N02-analgesics. The number of live births was an ordinal variable with five groups – none, one, two, three, four or more live births. Smoking habits (never, ex and current smoker) and alcohol drinking (never, social occasion, weekly) were nominal variables. At year 20, smoking and alcohol drinking were not collected, and menopause status was assumed postmenopausal.

### 2.6 Statistical analysis

We provided study sample's descriptive statistics at both follow-ups. Our main analyses involved three steps: (1) identification of BMI trajectories; (2) investigation of baseline musculoskeletal pain types as predictors of BMI trajectory-groups; (3) assessment of the association between BMI trajectory-groups and distal outcomes – year 20 musculoskeletal pain and mortality.

We used group-based trajectory modelling to identify latent clusters of women whose BMI follows the same pattern over follow-up time [16]. We fitted censored normal models with up to forth polynomial [17]. The best fit was determined using Bayesian Information Criteria and group posterior probabilities (>0.70), as previously described [10,17,18]. To conclude whether defined BMI trajectory-groups were distinct or parallel, we compared intercepts and the same order polynomial coefficients using the Wald test [10,19]. Further, to investigate whether single- and multi-site musculoskeletal pain at baseline was associated with BMI trajectories, we used multinomial logistic regression. To assess whether 19-year BMI trajectory-groups were predictors of musculoskeletal pain in year 20, we used binary logistic regression. Finally, to determine whether BMI trajectories were associated with all-cause and cause-specific mortality, we used Cox proportional-hazards models. We plotted the cumulative hazard function of all-cause mortality per BMI trajectory-group. All models were adjusted for the confounding variables.

Although baseline variables were mostly complete, there were missing values during, and at the end of the follow-up. Group-based trajectory modelling using maximum likelihood handle subjects with missing data at random, and it is suitable for understanding the nature/pattern of non-random attrition [18,20]. To account for missing information in covariates in year 20, we used multiple imputations fully conditional specification method with ten imputed datasets and reported pooled estimates. We did not impute BMI or musculoskeletal pain. We used SAS 9.4 (SAS Institute, Cary, North Caroline), proc traj package, macros trajtest and trajplotnew (https://www.andrew.cmu.edu/user/bjones/) and SPSS Statistics 27 (IBM, Chicago, Illinois).

## 3 Results

Of 1003 Chingford women, we included 938 (93.5%) women. We excluded 41 (4.1%) women with one and 24 (2.4%) with two BMI measures. Of included women, 520 (51.8%) had ten or 11 BMI measures. In year 20, 566 (60.3%) women attended the follow-up. Percentages of missing values in year 20 covariates ranged from 12.7% to 16.3%, and these were imputed.

In year 1, women were on average 54 years old (SD=5.99), mostly menopausal (76.4%), never smoked (54.4%), and drank alcohol on social occasions (43.4%). In year 20, women were on average 72 years old (SD=5.56). Percentages of time-stable covariates, the number of live births and oral contraceptive pill use, were almost the same in women who participated in both follow-ups. However, in year 20, women reported analgesic use six times more often, cancer four times, fractures and orthopedic operations approximately three times, and other major illnesses twice as often as in year 1. Overall mean BMI increased from 25.5 to 27.8 kg/m^2^. Although the prevalence of single-site musculoskeletal pain was almost the same at both follow-ups, the prevalence of two and three painful sites doubled and tripled in the year 20, respectively. The most commonly reported pain in year 1 was back pain (51.8%), and in year 20, hand (50.2%), and knee (49.8%) pain. Table 1 shows details of the study sample.

**Table 1:** Descriptive statistics of the study sample.

*Variable*	Follow-up Year 1 N = 938^a^	Follow-up Year 20 N = 566^b^
*Age* (years), mean (SD)	54.14 (5.99)	72.11 (5.56)
*Menopause status*, % Menopaused	76.4	N/A
*Number of live births*, % None One Two Three Four and more	13.2 15.5 39.6 19.7 12.0	12.4 13.3 45.6 19.8 9.0
*Smoking habits*, % Never smoked Ex-smoker Current smoker	54.4 23.1 22.5	N/A
*Alcohol drinking*, % Never Social occasions Weekly	18.6 43.4 38.1	N/A
*Physical activity, %* Active Walking Sport Job	91.2 47.8 20.0 85.7	97.5 81.4 12.2 96.3
*Oral contraceptive pill use (ever)*, % Yes	33.3	34.2
*Hormone replacement therapy use (ever)*, % Yes	24.1	38.9
*Analgesic use*, % Yes	7.3	44.3
*Hysterectomy*, % Yes	23.0	33.4
*Cancer*, % Yes	4.2	17.7
*Fractures in last 10 years*, % Yes	11.8	33.3
*Orthopedic operations (ever)*, % Yes	9.3	25.7
*Other major illnesses*, % Yes	24.3	47.3
*Body mass index (kg/m^2^),* mean (SD)	25.51 (4.15)	27.80 (4.90)
*Musculoskeletal pain,* single site % Any single site Back pain Hand pain Hip pain Knee pain	72.4 51.8 31.1 N/A 30.6	73.7 34.9 50.2 31.8 49.8
*Musculoskeletal pain,* multi-site *%* Any two sites Back and hand Back and hip Back and knee Hand and hip Hand and knee Hip and knee All/Any three sites Back, hand and hip Back, hand and knee Back, hip and knee Hand, hip and knee All four sites	32.5 9.1 N/A 8.7 N/A 14.7 N/A 8.6 N/A 8.6 N/A N/A N/A	57.7 3.7 2.0 2.5 6.1 19.6 23.9 30.1 3.5 5.4 3.3 17.8 10.2

SD, standard deviation; N/A, not applicable or not available.

a There were 12 women with missing values; one woman with a missing value in menopause, seven with missing values in physical activity, another one with analgesic use, two women with a missing value in cancer, and one with missing values in cancer and other major illness variables.

b At year 20 body mass index and musculoskeletal pain were not complete or imputed. Body mass index was available for 515 women, back pain for 478, hand pain for 558, hip pain for 557, and knee pain for 564 women. The percentage for multiple sites musculoskeletal pain was based on 511 women for two sites, 482 for three sites, and 470 women for complete musculoskeletal pain data.

### 3.1 BMI trajectories

**Fig. 1:**
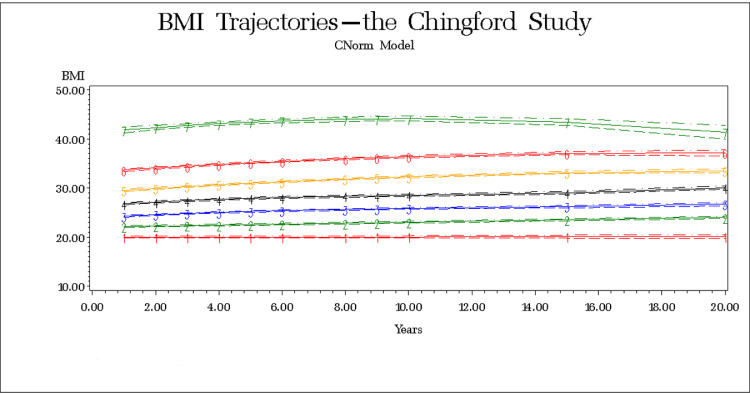
Body mass index trajectories; groups 1 and 2 - normal-weighted and used together as a reference, group 3 - slightly overweight, group 4 - lower overweight-to-obese, group 5 - upper overweight-to-obese, group 6 - lower obese, and group 7 - upper obese.

We identified seven BMI trajectories (Table 2, Fig. 1) and named them considering the WHO categories. The goodness-of-fit is shown in Supplement. Posterior probabilities of group membership ranged from 0.94 to 1.00, indicating the excellent classification. The Wald test results showed all trajectories had significantly different intercepts. Other parameters were compared between the same order curves; the linear curves (groups 1 and 2) had different patterns/slopes; cubic curves (groups 3 and 4) were parallel, the same as quadratic (groups 5 and 6), and group 7 had a different pattern from groups 5 to 6.

**Table 2:** Body mass index trajectories.

Trajectory-group	Curve order	Curve parameters				*Group size, N (%)*	*Posterior probability*
		Intercept	Linear	Quadratic	Cubic		
1	1	20.02	0.0065	N/A		68 (7.2)	0.96
2	1	22.10	0.0948	N/A		216 (23.0)	0.96
3	3	23.94	0.3509	-0.0217	0.0006	260 (27.7)	0.94
4	3	26.36	0.4534	-0.0336	0.0010	205 (21.9)	0.95
5	2	29.18	0.4178	-0.0096	N/A	118 (12.6)	0.97
6	2	33.25	0.4380	-0.0122	N/A	59 (6.3)	0.99
7	2	41.29	0.5606	-0.0278	N/A	12 (1.3)	1.00

N/A, not applicable. The model was created using 11 repeated measures of body mass index at follow-up years 1, 2, 3, 4, 5, 6, 8, 9, 10, 15 and 20. Group 1 and 2 are normal-weighted and used together as a reference. The remaining five groups are labelled as slightly overweight (group 3), lower overweight-to-obese (group 4), upper overweight-to-obese (group 5), lower obese (group 6), and upper obese (group 7).

Because 95% confidence intervals of the second group at all time points stayed below 25 (Supplement), we combined groups 1 and 2 into the normal-weighted reference. BMI of 24-27 characterized group 3 – slightly overweight. Women in group 4 - lower overweight-to-obese had BMI 27-30, and those in group 5 – upper overweight-to-obese 29-34. Obese women were clustered into lower (group 6) with BMI 33-38 and upper (group 7) obese with BMI 41-43.

Baseline characteristics of BMI trajectories are in Appendix. Briefly, women in upper overweight-to-obese and upper obese groups more often reported use of analgesics, while lower and upper overweight-to-obese and upper obese fractures, single and multi-site musculoskeletal pain.

### 3.2 BMI trajectories and musculoskeletal pain

We assessed whether women's pain reports at baseline could predict BMI trajectory over the next 19 years. We found that women who reported back pain and knee pain at baseline had increased odds of having lower and upper overweight-to-obese and upper obese trajectories. Also, women reporting two, and three painful sites had a significantly increased risk of both overweight-to-obese patterns.

Further, we assessed whether woman's 19-year BMI trajectory was associated with an increased risk of musculoskeletal pain in year 20. We found that women had increased odds of experiencing back, hand, hip or knee pain when their BMI trajectory was lower or upper overweight-to-obese or lower obese. These three trajectories were associated with increased odds of multi-site musculoskeletal pain (Table 3).

**Table 3:** Body mass index trajectories and single and multi-site musculoskeletal pain.

*Predictor*	*Outcome*				
**Follow-up Year 1**					
MSK pain	BMI trajectories^a^*OR (95%CI)*				
	*Slightly overweight*	*Lower overweight-to-obese*	*Upper overweight-to-obese*	*Lower obese*	*Upper obese*
*Back pain*	1.33 (0.93-1.90)	**1.58** (1.08-2.32)	1.40 (0.89-2.22)	1.20 (0.66-2.18)	2.51 (0.68-9.28)
*Hand pain*	0.84 (0.57-1.25)	1.16 (0.77-1.74)	1.08 (0.66-1.76)	0.81 (0.42-1.56)	0.99 (0.27-3.59)
*Knee pain*	1.18 (0.79-1.77)	**1.93** (1.28-2.93)	**2.55** (1.58-4.11)	1.49 (0.78-2.82)	**3.39** (1.00-11.49)
*Two sites*	1.00 (0.68-1.49)	**1.54** (1.03, 2.32)	**1.75** (1.08, 2.83)	1.04 (0.54, 2.01)	1.64 (0.46, 5.81)
*Three sites*	1.89 (0.93, 3.85)	1.68 (0.78, 3.63)	**3.21** (1.48, 6.96)	1.78 (0.62, 5.15)	3.75 (0.68, 20.70)

**Follow-up Year 20**							
	MSK pain^b^*OR (95% CI)*						
BMI trajectories	*Back pain*	*Hand Pain*	*Hip pain*	*Knee pain*	*Two sites*	*Three sites*	*Four sites*
*Slightly overweight*	0.95 (0.55, 1.63)	1.52 (0.96, 2.41)	1.37 (0.82, 2.30)	1.46 (0.92, 2.34)	1.28 (0.78, 2.12)	1.70 (0.94, 3.09)	1.36 (0.55, 3.35)
*Lower overweight-to-obese*	1.20 (0.68, 2.12)	**2.14** (1.30, 3.51)	1.58 (0.91, 2.74)	**1.77** (1.07, 2.92)	1.58 (0.93, 2.70)	**2.58** (1.39, 4.79)	1.60 (0.63, 4.10)
*Upper overweight-to-obese*	**3.14** (1.57, 6.29)	**2.08** (1.14, 3.80)	1.92 (0.99, 3.68)	**2.07** (1.13, 3.81)	**2.94** (1.46, 5.92)	**3.25** (1.56, 6.78)	**2.85** (1.00, 8.12)
*Lower obese*	2.19 (0.76, 6.32)	1.30 (0.56, 3.01)	**2.58** (1.03, 6.41)	**2.72** (1.12, 6.61)	1.96 (0.72, 5.29)	**3.92** (1.37, 11.20)	2.03 (0.45, 9.14)
*Upper obese*	0.85 (0.06, 11.25)	-	7.56 (0.65, 87.59)	2.64 (0.23, 30.71)	-	5.29 (0.33, 84.17)	4.52 (0.31, 66.61)

BMI, body mass index; MSK, musculoskeletal; OR, odds ratio; CI, confidence interval.

a The number of observations in these models was 926 (per BMI trajectory-group: normal weight 277, then from slightly overweight to upper obese, 257, 204, 118, 58, 12, respectively). The models were created using nominal regression models with reference group normal weight (BMI trajectory groups 1 and 2) and adjusted for age, menopause status, the number of live births, smoking habits, alcohol drinking, physical activity, oral contraceptive pill use, hormone replacement therapy use, analgesic use, hysterectomy, cancer, fractures, orthopedic operations, and other major illness.

b The number of observations in back pain model was 478 (per BMI trajectory-group: normal weight to upper obese, 148, 141, 113, 55, 18, 3, respectively); in hand pain 558 (per BMI trajectory-group: normal weight to upper obese, 175, 160, 124, 67, 28, 4, respectively); in hip pain 557 (per BMI trajectory-group: normal weight to upper obese, 176, 158, 124, 68, 27, 4, respectively); in knee pain 564 (per BMI trajectory-group: normal weight to upper obese, 178, 162, 123, 69, 28, 4, respectively); in two musculoskeletal pain sites 511 (per BMI trajectory-group: normal weight to upper obese, 154, 149, 118, 62, 24, 4, respectively); in three sites 482 (per BMI trajectory-group: normal weight to upper obese, 146, 140, 115, 57, 21, 3, respectively); and 470 in four sites model (per BMI trajectory-group: normal weight to upper obese, 144, 138, 113, 54, 18, 3, respectively). The models were created using binary logistic regression estimating odds of single painful site vs. no pain, two painful sites vs. single painful site or no pain, three painful sites vs. two, one or none painful site, and four vs. three, two, one or none painful site. All models were adjusted for age, the number of live births, physical activity, oral contraceptive pill use, hormone replacement therapy use, analgesic use, hysterectomy, cancer, fractures, orthopedic operations, and other major illness. Significant results are bolded.

### 3.3 BMI trajectories and mortality

**Fig. 2:**
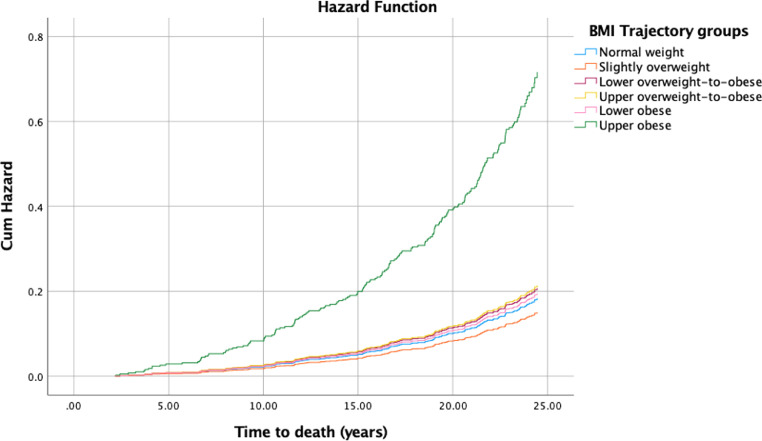
Cumulative hazard function of all-cause mortality for body mass index trajectories.

The cumulative number of all deaths over the 25-year follow-up (median 23.6 years, interquartile range 23.1-24.3) was 201 (21.4%). The cardiovascular cause occurred in 53 (26.4%) and cancer cause in 92 (45.8%) cases. We found that women in the upper obese trajectory-group had a four-time increased hazard of all-cause mortality and almost six-time of cardiovascular mortality compared to normal-weighted women (Table 4, Fig. 2).

**Table 4:** Body mass index trajectories and mortality.

*BMI trajectory*	*All cause death*^a^		*Cardiovascular-related death*^a^		*Cancer-related death*^a^	
	*N*	*HR (95% CI)*	*N*	*HR (95% CI)*	*N*	*HR (95% CI)*
Slightly overweight	47	0.82 (0.55, 1.23)	12	0.93 (0.42, 2.08)	22	0.85 (0.47, 1.53)
Lower overweight-to-obese	49	1.13 (0.76, 1.68)	10	0.86 (0.37, 2.01)	23	1.25 (0.70, 2.24)
Upper overweight-to-obese	29	1.17 (0.73, 1.88)	10	1.53 (0.64, 3.66)	12	1.22 (0.60, 2.50)
Lower obese	14	1.06 (0.58, 1.95)	6	1.83 (0.66, 5.12)	5	0.87 (0.33, 2.32)
Upper obese	6	**3.90** (1.60, 9.49)	2	**5.47** (1.11, 26.83)	3	3.52 (0.99, 12.61)

BMI, body mass index; N, number of events; HR, hazard ratio; CI, confidence interval.

a The number of observations in these models was 926 (per BMI trajectory-group: normal weight 277, then from slightly overweight to upper obese, 257, 204, 118, 58, 12, respectively). The models were created using Cox proportional hazards model with reference group normal weight (BMI trajectory groups 1 and 2) and adjusted for age, menopause status, the number of live births, smoking habits, alcohol drinking, physical activity, oral contraceptive pill use, hormone replacement therapy use, analgesic use, hysterectomy, cancer, fractures, orthopedic operations, and other major illness at baseline. Significant results are bolded.

## 4 Discussion

In UK postmenopausal women, we found seven distinct 19-year BMI patterns, relatively steady in normal-weighted but increasing over time in overweight, and obese women. We observed a consistent bidirectional relationship between lower and upper overweight-to-obese BMI patterns with knee pain and upper overweight-to-obese with multi-site musculoskeletal pain. Women with upper obese 19-year BMI trajectory had an increased risk of all-cause and cardiovascular mortality over normal-weighted women.

This study was done in a population-representative sample receiving the same health care (GP practice and research nurse), using measured not self-reported weight, and height over two decades of the critical life period when different pathologies onset. However, there are some limitations to be considered. The findings are related to women only. The pain questions slightly differed per assessment and did not reflect the definition of chronic pain (duration of symptoms), except year 20 back pain. However, the pain episode irrespective of chronicity is of great public health importance, and severity was captured by spread, i.e., multi-site pain. Further, we did not consider the underlying pathology of musculoskeletal pain. The joint diseases, osteoarthritis being the most common among them, are defined mostly based on the symptom/pain with inconsistent, and weak evidence for the importance of structural changes [21,22]. Thus, our study focused on pain – the essential patient-reported outcome, the primary descriptor of their impaired quality of life, and the main determinant of the treatment. Finally, despite accounting for numerous confounding, we cannot rule out the residual confounding.

We identified seven BMI trajectories described by different intercepts and 19-year patterns. We found two normal-weighted groups (30.2%). Interestingly, women with BMI around 20 had a minimal change (the slope almost zero) over two decades. The rest of normal-weighted women had a steady BMI between 22 and 24. Three of our groups that account for 62.2% of the sample were changing the WHO categories. The largest observed group - slightly overweight, included 27.7% of women whose BMI fluctuated around 25. In the first few years of the follow-up, women were normal-weighted, and then in their late 50s became overweight. We found two groups of overweight women who became obese over time. Women in the lower overweight-to-obese group had BMI around 27 at the baseline and shifted to the WHO obese category in their 70s. However, women in the upper overweight-to-obese group were just below the obesity cut-off in the first years and obese further on. In our sample, we detected only 7.6% of women who were obese all the observational time, and only 1.3% had a BMI above 40 (upper obese group). The previous reports from the US Health and Retirement Study [23], that started in the early 1990s with women and men at baseline aged 55, used the same methodology and reported remarkably similar findings over a 14-year follow-up. They found five BMI trajectories with intercepts and patterns almost exactly like in our second normal-weighted, lower and upper overweight-to-obese, lower and upper obese groups [23]. Long-term BMI patterns in middle-aged individuals seem comparable across nations.

Several reports investigated BMI trajectories slightly differently but assessed the risk of mortality [24], [25], [26], [27]. The association between single-time BMI and mortality has been known as the obesity paradox, i.e., overweight people have the lowest mortality risk [28,29]. Our findings are similar to previous studies in people aged 40 or 50 at baseline and followed-up until their mid-sixties that detected six BMI trajectories [26,27]. The US study found increased mortality in obese and normal-decreasing trajectories [27]. The Australian study reported increased risks for all-cause and cause-specific mortality in all obese groups [26]. Our study is the first report on BMI trajectories in UK women aged on average 54 years at baseline and mortality data collected over 25 years. Notably, in 1989, for UK women, life expectancy at age 54 was 26.8 years [30]. None of the previous studies consider life expectancy in their follow-up duration. We found that only women with BMI above 40 were unlikely to meet population life expectancy, due to the increased all-cause and cardiovascular mortality risk. Thus, the findings of the obese trajectory, although the smallest group, and mortality are reproducible and consistent across the nations.

Importantly, this is the first report on bidirectional relationships between long-term BMI patterns and musculoskeletal pain. In our sample, the prevalence of any single site pain was high, around 70% at both follow-ups, although multi-site pain became more prevalent with age, and more than half of women in their 70s reported two-site pain and almost a third three-site pain. In year 20, we had hip pain that could account for some difference in multi-site prevalence although it did not affect single-site prevalence. Prominently, knee pain was bi-directionally associated with lower and upper overweight-to-obese trajectories, highlighting women having BMI 27-34 as the highest risk group with obesity and pain influencing each other. It means that knee pain contributed to their weight gain, and also becoming obese over time increased the odds of experiencing pain later on, creating a vicious circle. This group could benefit the most from weight reduction management of pain, but it is also likely that due to pain they do not engage in this treatment option. Indeed, most studies in patients with knee OA reported average BMI within this range [9,10,31]. Furthermore, these two BMI trajectories were unidirectionally related to back and hand pain. Taken together, it narrowed and emphasized the consistent relationship between the upper overweight-to-obese trajectory (BMI 29-34) with multi-site musculoskeletal pain. The lower obese pattern over 19 years was associated with pain in lower limbs – hip and knee. Because of the competing mortality risk, women in upper obese group were unlikely to report year 20 pain. Notably, slightly overweight women had no increased risk of any musculoskeletal pain or mortality compared to normal-weighted women.

To conclude, we showed 19-year BMI patterns among postmenopausal UK women, allowing us to recognize the WHO BMI category change overtime. We found that women with BMI 27-34 were most likely to experience musculoskeletal pain, although those with BMI above 40 were at increased risk of all-cause and cardiovascular mortality. However, women whose BMI was around 25, but not above 27, had no different risk of pain or mortality than normal-weighted women. BMI of 27 could be an initial goal for weight reduction concerning musculoskeletal pain, and it might increase patients’ motivation, engagement and compliance for achieving an easier target although reducing the risk. Also, future research on BMI and pain should go beyond the WHO categories and consider the risk group and bidirectional relationship identified here for prevention and management of musculoskeletal pain.

## Role of the funder/sponsor

The funding sources had no role in the design and conduct of the study; collection, management, analysis, and interpretation of the data; preparation, review, or approval of the manuscript; and decision to submit the manuscript for publication.

## Acknowledgements

We would like to thank all the participants of the Chingford 1,000 Women Study, Dr Alan Hakim (The Wellington Hospital) study coordinator, Ms Maxine Daniels (Chingford Hospital) research nurse, and Ms Alison Turner (University of Oxford) data manager, for their time and dedication. We would like to thank Ms Julie Damnjanović (University of Oxford) for her valued support to the team and the study data management.

## Appendix Supplementary materials

Image, application 1
