# Supplementary material for: Specific body mass index trajectories were related to musculoskeletal pain and mortality: 19‐year follow‐up cohort
Source: J Clin Epidemiol. 2022 Jan;141:54–63. doi: 10.1016/j.jclinepi.2021.09.020 (PMC8982643; doi:10.1016/j.jclinepi.2021.09.020)
Supplement: Supplementary file 1 [file mmc1.docx]

| **Supplementary Table 1 Goodness-of-fit of the BMI trajectory modelling** | | | | |
| --- | --- | --- | --- | --- |
| *Number of groups** | *Order* | *Group percentage* | *Bayesian Information Criterion* | *Posterior probability* |
| 1 | 4 | 100 | -25153.88 | 1.00 |
| 1 | 3 | 100 | -25150.54 | 1.00 |
| 2 | 3 3 | 69.1; 30.9 | -22077.69 | 0.99; 0.97 |
| 3 | 3 3 3 | 47.6; 40.2; 12.4 | -20398.75 | 0.98; 0.97; 0.99 |
| 3 | 1 3 3 | 47.4; 40.3; 12.3 | -20394.22 | 0.98; 0.97; 0.99 |
| 4 | 3 3 3 3 | 32.3; 41.8; 21.2; 4.7 | -19308.53 | 0.98; 0.97; 0.98; 0.99 |
| 4 | 1 3 2 3 | 32.3; 41.8; 21.2; 4.7 | -19300.12 | 0.98; 0.97; 0.98; 0.99 |
| 5 | 1 3 3 3 2 | 26.3; 36.8; 23.8; 11.2; 1.9 | -18514.67 | 0.97; 0.97; 0.97; 0.98; 0.99 |
| 6 | 1 3 3 2 3 2 | 18.8; 30.8; 25.7; 16.0; 7.3; 1.5 | -18004.04 | 0.97; 0.95; 0.95; 0.97; 0.99; 0.99 |
| 7 | 1 1 3 3 2 2 2 | 7.3; 23.0; 27.7; 21.9; 12.6; 6.3; 1.3 | -17633.24 | 0.96; 0.96; 0.94; 0.95; 0.97; 0.99; 1.00 |
| ^*^The eight-group models could not provide all parameter estimates and standard errors; thus, modelling ended with the best fit seven-group model. | | | | |

| **Supplementary Table 2 Predicted BMI estimates from the best fit model ^a^** | | | | | | | | | | |  |
| --- | --- | --- | --- | --- | --- | --- | --- | --- | --- | --- | --- |
|  | *Year 1* | *Year 2* | *Year 3* | *Year 4* | *Year 5* | *Year 6* | *Year 8* | *Year 9* | *Year 10* | *Year 15* | *Year 20* |
| *Trajectory-group* | *Predicted BMI*  *Mean (95% Confidence Interval)* | | | | | | | | | | |
| 1 | 20.03  (19.80, 20.26) | 20.03  (19.82, 20.25) | 20.04  (19.84, 20.24) | 20.05  (19.85, 20.24) | 20.05  (19.87, 20.24) | 20.06  (19.88, 20.24) | 20.07  (19.89, 20.26) | 20.08  (19.89, 20.27) | 20.09  (19.89, 20.29) | 20.12  (19.84, 20.40) | 20.15  (19.76, 20.54) |
| 2 | 22.20  (22.06, 22.34) | 22.29  (22.16, 22.43) | 22.39  (22.26, 22.51) | 22.48  (22.36, 22.60) | 22.58  (22.46, 22.70) | 22.67  (22.55, 22.79) | 22.86  (22.74, 22.98) | 22.96  (22.83, 23.08) | 23.05  (22.92, 23.18) | 23.52  (23.35, 23.70) | 24.00  (23.76, 24.23) |
| 3 | 24.27  (24.08, 24.46) | 24.56  (24.40, 24.72) | 24.81  (24.66, 24.96) | 25.03  (24.87, 25.19) | 25.22  (25.06, 25.39) | 25.38  (25.22, 25.55) | 25.64  (25.47, 25.81) | 25.74  (25.57, 25.92) | 25.83  (25.65, 26.01) | 26.19  (25.94, 26.44) | 26.71  (26.38, 27.03) |
| 4 | 26.78  (26.56, 27.00) | 27.14  (26.96, 27.32) | 27.45  (27.27, 27.62) | 27.70  (27.52, 27.88) | 27.92  (27.73, 28.10) | 28.09  (27.89, 28.29) | 28.36  (28.14, 28.57) | 28.46  (28.24, 28.68) | 28.55  (28.31, 28.78) | 29.02  (28.72, 29.32) | 30.09  (29.76, 30.43) |
| 5 | 29.58  (29.32, 29.83) | 29.96  (29.73, 30.18) | 30.32  (30.11, 30.52) | 30.66  (30.47, 30.84) | 30.98  (30.79, 31.16) | 31.28  (31.09, 31.47) | 31.83  (31.62, 32.03) | 32.08  (31.86, 32.28) | 32.30  (32.09, 32.51) | 33.14  (32.89, 33.39) | 33.51  (33.06, 33.95) |
| 6 | 33.67  (33.38, 33.97) | 34.07  (33.84, 34.31) | 34.45  (34.25, 34.65) | 34.81  (34.62, 34.99) | 35.13  (34.95, 35.32) | 35.44  (35.25, 35.63) | 35.97  (35.75, 36.20) | 36.21  (35.97, 36.44) | 36.41  (36.16, 36.66) | 37.08  (36.77, 37.40) | 37.15  (36.54, 37.75) |
| 7 | 41.83  (41.20, 42.45) | 42.30  (41.81, 42.80) | 42.72  (42.32, 43.13) | 43.09  (42.73, 43.45) | 43.40  (43.04, 43.76) | 43.66  (43.27, 44.04) | 44.00  (43.54, 44.46) | 44.09  (43.60, 44.57) | 44.12  (43.61, 44.63) | 43.45  (42.77, 44.13) | 41.39  (40.00, 42.78) |
| ^a^ The predicted estimates shown here are graphically presented in Figure 1. | | | | | | | | | | | |

| **Supplementary Table 3 Baseline descriptive statistics of the BMI trajectory groups** | | | | | | | |
| --- | --- | --- | --- | --- | --- | --- | --- |
| *Variable* | BMI group 1  N=68 | BMI group 2  N=216 | BMI group 3  N=260 | BMI group 4  N=205 | BMI group 5  N=118 | BMI group 6  N=59 | BMI group 7  N=12 |
|  | *Reference* | | *Slightly overweight* | *Lower overweight-to-obese* | *Upper overweight-to-obese* | *Lower obese* | *Upper obese* |
| *Age* (years), mean (SD) | 53.29 (6.52) | 53.36 (6.02) | 54.38 (5.88) | 54.69 (5.92) | 54.52 (5.96) | 54.22 (6.01) | 54.58 (5.38) |
| *Menopause status*, %  Menopaused | 69.1 | 74.0 | 78.1 | 81.5 | 75.4 | 71.2 | 75.0 |
| *Number of live births*, %  None  One  Two  Three  Four and more | 19.1  19.1  26.5  26.5  8.8 | 19.0  14.8  43.1  14.8  8.3 | 10.8  13.1  45.8  17.3  13.1 | 9.3  16.1  38.0  22.9  13.7 | 11.9  17.8  33.1  22.0  15.3 | 10.2  16.9  37.3  22.0  13.6 | 25.0  16.7  16.7  33.3  8.3 |
| *Smoking habits*, %  Never smoked  Ex-smoker  Current smoker | 57.4  13.2  29.4 | 50.9  23.1  25.9 | 55.8  21.9  22.3 | 54.1  25.9  20.0 | 50.0  29.7  20.3 | 67.8  15.3  16.9 | 50.0  33.3  16.7 |
| *Alcohol drinking*, %  Never  Social occasions  Weekly | 14.7  44.1  41.2 | 20.8  40.3  38.9 | 15.8  41.9  42.3 | 17.6  42.4  40.0 | 16.9  54.2  28.8 | 30.5  44.1  25.4 | 33.3  33.3  33.4 |
| *Physical activity, %*  Active  Walking  Sport  Job | 88.1  46.3  20.9  81.8 | 90.1  52.1  23.0  85.4 | 90.3  47.3  24.9  84.1 | 94.1  49.8  20.5  87.8 | 89.8  42.4  10.2  83.9 | 94.8  44.8  6.9  93.1 | 91.7  25.0  8.3  91.7 |
| *Oral contraceptive pill use (ever)*, %  Yes | 30.9 | 38.9 | 33.1 | 30.2 | 32.2 | 30.5 | 25.0 |
| *Hormone replacement therapy use (ever)*, %  Yes | 19.1 | 24.5 | 25.0 | 22.9 | 28.8 | 20.3 | 16.7 |
| *Analgesic use*, %  Yes | 6.0 | 4.6 | 7.3 | 5.9 | 13.6 | 8.5 | 16.7 |
| *Hysterectomy*, %  Yes | 19.1 | 21.3 | 21.2 | 24.4 | 31.4 | 22.0 | 16.7 |
| *Cancer*, %  Yes | 5.9 | 2.8 | 3.9 | 4.4 | 5.9 | 3.4 | 8.3 |
| *Fractures in last 10 years*, %  Yes | 8.8 | 10.6 | 8.5 | 16.6 | 14.4 | 8.5 | 33.3 |
| *Orthopaedic operations (ever)*, %  Yes | 7.4 | 6.5 | 10.4 | 10.7 | 7.6 | 15.3 | 8.3 |
| *Other major illnesses*, %  Yes | 25.0 | 23.3 | 25.0 | 21.5 | 26.3 | 30.5 | 25.0 |
| *Musculoskeletal pain,* single site %  Any single site  Back pain  Hand pain  Knee pain | 61.8  47.1  30.9  25.0 | 66.2  44.9  28.2  22.2 | 70.4  53.5  28.8  26.5 | 82.0  56.6  34.6  36.1 | 77.1  55.1  36.4  45.8 | 69.5  49.2  28.8  32.2 | 91.7  66.7  33.3  50.0 |
| *Musculoskeletal pain,* multiple sites *%*  Any two sites  Back and hand  Back and knee  Hand and knee  All three sites | 33.8  10.3  13.2  10.3  7.4 | 25.0  8.8  6.0  10.2  4.2 | 29.2  7.3  7.7  14.2  9.2 | 37.6  11.7  9.3  16.6  7.8 | 44.1  10.2  11.9  22.0  16.1 | 30.5  6.8  8.5  15.3  10.2 | 41.7  0.0  16.7  25.0  16.7 |
| BMI – body mass index; SD – standard deviation; N/A – not applicable or not available.  ^a^ There were 12 women with missing values; the woman with a missing value in menopause was in the BMI group 2, missing values in physical activity had a woman in BMI group 1, three women in BMI group 2, two women in BMI group 3 and a woman in BMI group 6, another one with analgesic use in the BMI group 1, two women with a missing value in cancer were in BMI group 3 and 4, and one with missing values in cancer and other major illness was in the BMI group 2. | | | | | | | |
